# Grb2 binds to PTEN and regulates its nuclear translocation to maintain the genomic stability in DNA damage response

**DOI:** 10.1038/s41419-019-1762-3

**Published:** 2019-07-18

**Authors:** Bolin Hou, Shanshan Xu, Yang Xu, Quan Gao, Caining Zhang, Ling Liu, Huaiyi Yang, Xuejun Jiang, Yongsheng Che

**Affiliations:** 1 State Key Laboratory of Mycology, Institute of Microbiology, Chinese Academy of Sciences, 100101 Beijing, China; 2 University of Chinese Academy of Sciences, 100039 Beijing, China; 3 Institute of Medicinal Biotechnology, Chinese Academy of Medical Sciences & Peking Union Medical College, 100050 Beijing, China; 4 CAS Key Laboratory of Pathogenic Microbiology and Immunology, Institute of Microbiology, Chinese Academy of Sciences, 100101 Beijing, China

**Keywords:** Growth factor signalling, Nuclear receptors

## Abstract

Growth factor receptor bound protein 2 (Grb2) is an adaptor protein critical for signal transduction and endocytosis, but its role in DNA damage response (DDR) remains unknown. Here, we report that either knockdown of Grb2 or overexpression of the mutated Grb2 promotes micronuclei formation in response to oxidative stress. Furthermore, Grb2 was demonstrated to interact with phosphatase and tensin homologue (PTEN; a tumor suppressor essential for nuclear stability), and the loss of Grb2 reduced the nuclear-localized PTEN, which was further decreased upon stimulation with hydrogen peroxide (H_2_O_2_). Overexpression of the T398A-mutated, nuclear-localized PTEN reduced micronuclei frequency in the cells deficient of functional Grb2 via rescuing the H_2_O_2_-dependent expression of Rad51, a protein essential for the homologous recombination (HR) repair process. Moreover, depletion of Grb2 markedly decreased the expression of Rad51 and its interaction with PTEN. Notably, Rad51 showed a preference to immunoprecipation with the T398A-PTEN mutant, and silencing of Rad51 alone accumulated micronuclei concurring with decreased expression of both Grb2 and PTEN. Our findings indicate that Grb2 interacts with PTEN and Rad51 to regulate genomic stability in DDR by mediating the nuclear translocation of PTEN to affect the expression of Rad51.

## Introduction

Grb2 is a critical adaptor protein connecting the activated receptor tyrosine kinases (RTKs) to the downstream targets, and displays multiple functions in embryogenesis, cancer cell differentiation, and DNA synthesis^1,2^, and its reduction has been demonstrated to inhibit the function of phosphatase and tensin homologue (PTEN) and to activate Akt^3^. Both Akt and PTEN appear to be responsible for genomic instability and the compromised DNA repair^4,5^. As a tumor suppressing gene, PTEN was found to be mutated in a variety of solid tumor cells^6–8^. Although usually considered as a cytoplasm-localized protein, accumulating evidence has revealed that PTEN located in nucleus and regulated nuclear stability via interacting with CENP-C and facilitating the transcription of Rad51^4^. Either phosphorylation or ubiquitination affected the nuclear localization of PTEN, which mediated the DNA damage response (DDR) by regulating Rad52 sumoylation^9^. Moreover, direct protein-protein interaction was recently found between PTEN and Rad51^10^.

Many elements, including reactive oxygen species, ionizing radiation, and chemical toxicity, were reported to induce nuclear damage^11–13^. In mammalian cells, non-homologous end joining and homologous recombination (HR) are two principal mechanisms to repair the DNA double-strand breaks (DSB)^14,15^. Central to DSB repair by HR is Rad51, which promotes strand invasion and homologous pairing between the two DNA duplexes, and is involved in a complex network of the damage-sensing and the cell cycle checkpoint signaling pathways, besides its direct role in HR process.

Both Grb2 and PTEN are closely related to the Akt signaling involved in DNA repair and Rad51 expression^5,16,17^. Moreover, the EGFR inhibitor gefitinib (Iressa) was found to attenuate the rate of DSB repair^18^. As an inhibitor of extracellularly regulated protein kinases 1/2 (ERK1/2; a member of the MAPK family and a downstream kinase of Grb2)^19^, U0126 appeared to reduce the level of Rad51^20^. Therefore, Grb2 likely regulates DDR through Rad51.

Micronuclei, nuclear buds, and nucleoplasmic bridges are the biomarkers of genotoxic and chromosomal instabilities. Since micronuclei can also originate from the chromosomal fragments of nucleoplasmic bridges^21^, its formation is likely one of the final fates for genotoxic events^22^. It has been reported that miRNAs targeted Rad51 to enhance the chemosensitivity of cells^23^, which could be reflected by micronuclei formation, and our previous results showed that Rad51 silencing increased the frequency of micronuclei in HeLa cells^24^.

In this study, we have demonstrated that Grb2 interacted with PTEN to mediate its nuclear translocation and increased Rad51 expression in response to oxidative stress to maintain nuclear stability. In addition, Rad51 bound preferentially to the nuclear-localized PTEN, deprivation of which increased micronuclei formation, whereas Rad51 silencing alone markedly decreased the expression of Grb2 and PTEN and increased micronuclei frequency.

## Results

### H_2_O_2_ stimulates the formation of micronuclei and the expression of Rad51

**Fig. 1:**
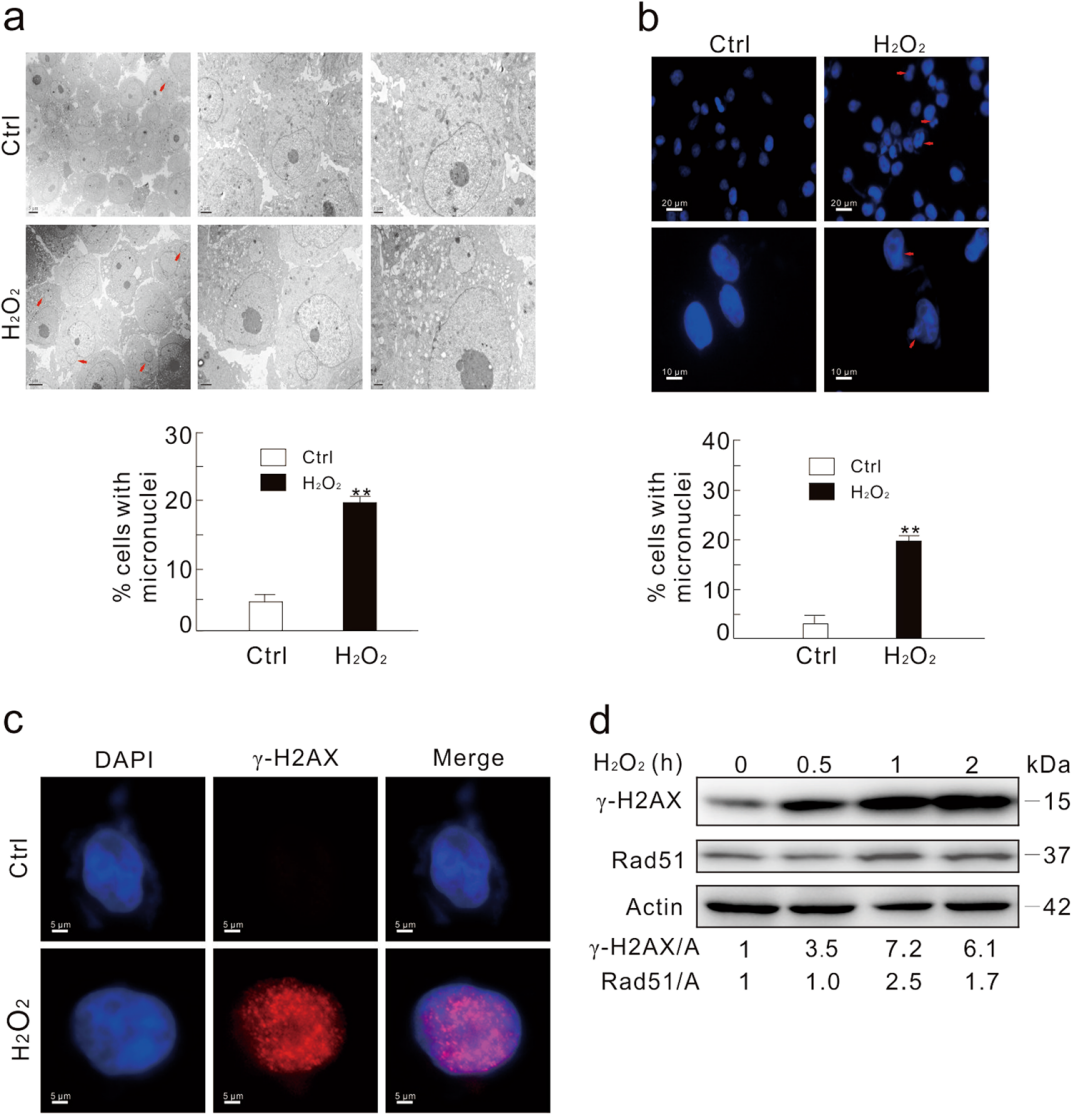
H_2_O_2_ increases micronuclei formation. **a** Electron microscopy was performed using the vehicle (Ctrl) or H_2_O_2_-treated (0.5 mM; 2 h) HeLa cells. The arrows indicate typical micronuclei. The number of cells containing micronuclei was counted and at least 30 cells were included in each group. **b** HeLa cells were treated with 0.5 mM H_2_O_2_ for 2 h, stained with DAPI, and observed with fluorescent microscope. The number of cells containing micronuclei was counted and at least 60 cells were included in each group. The data were normally distributed and statistically analyzed using the Studen–Newman–Keuls test. The double asterisks denote significant difference from control (**P < 0.01). **c** Immunofluorescence was performed using the γ-H2AX antibody in HeLa cells following treatment with 0.5 mM H_2_O_2_ for 2 h. **d** HeLa cells were treated with 0.5 mM H_2_O_2_ for the indicated times, and cell lysates were subjected to immunoblotting with the antibodies indicated. The adjusted ratios of γ-H2AX and Rad51 to actin (A) were presented below the blots. Similar experiments were repeated at least three times

Hydrogen peroxide (H_2_O_2_) is commonly used as a DNA damage inducer^25^. Under electron microscope (TEM), we observed that H_2_O_2_ increased micronuclei formation in HeLa cells (Fig. 1a), and micronuclei accumulation was actually initiated at the 0.5 h time point (Supplementary Fig. 1a). Cisplatin (CDDP), which was reported to arouse micronuclei^26^, also increased the micronuclei frequency (Supplementary Fig. 1b). Similar results were also obtained using fluorescence microscope (Fig. 1b). In addition, free micronuclei (60%), buds (40%), and bridges (20%) were independently quantified using EM and IF images in response to H_2_O_2_ treatment. For easy description, we combined these three for calculation and presented as micronuclei. In addition, foci of the phosphorylated histone H2AX (S139; γ-H2AX), a widely used marker for DNA double strands break (DSB)^27^, were elevated (Fig. 1c), and H_2_O_2_ increased γ-H2AX level in a time-dependent manner (Fig. 1d). Interestingly, the level of Rad51, a protein pivotal for DNA repair^28–30^, was also increased upon H_2_O_2_ challenge (Fig. 1d), suggesting that DNA repair may be concurrently triggered to eliminate the H_2_O_2_-induced nuclear damage.

### Depletion of Grb2 increases micronuclei formation upon H_2_O_2_ challenge

**Fig. 2:**
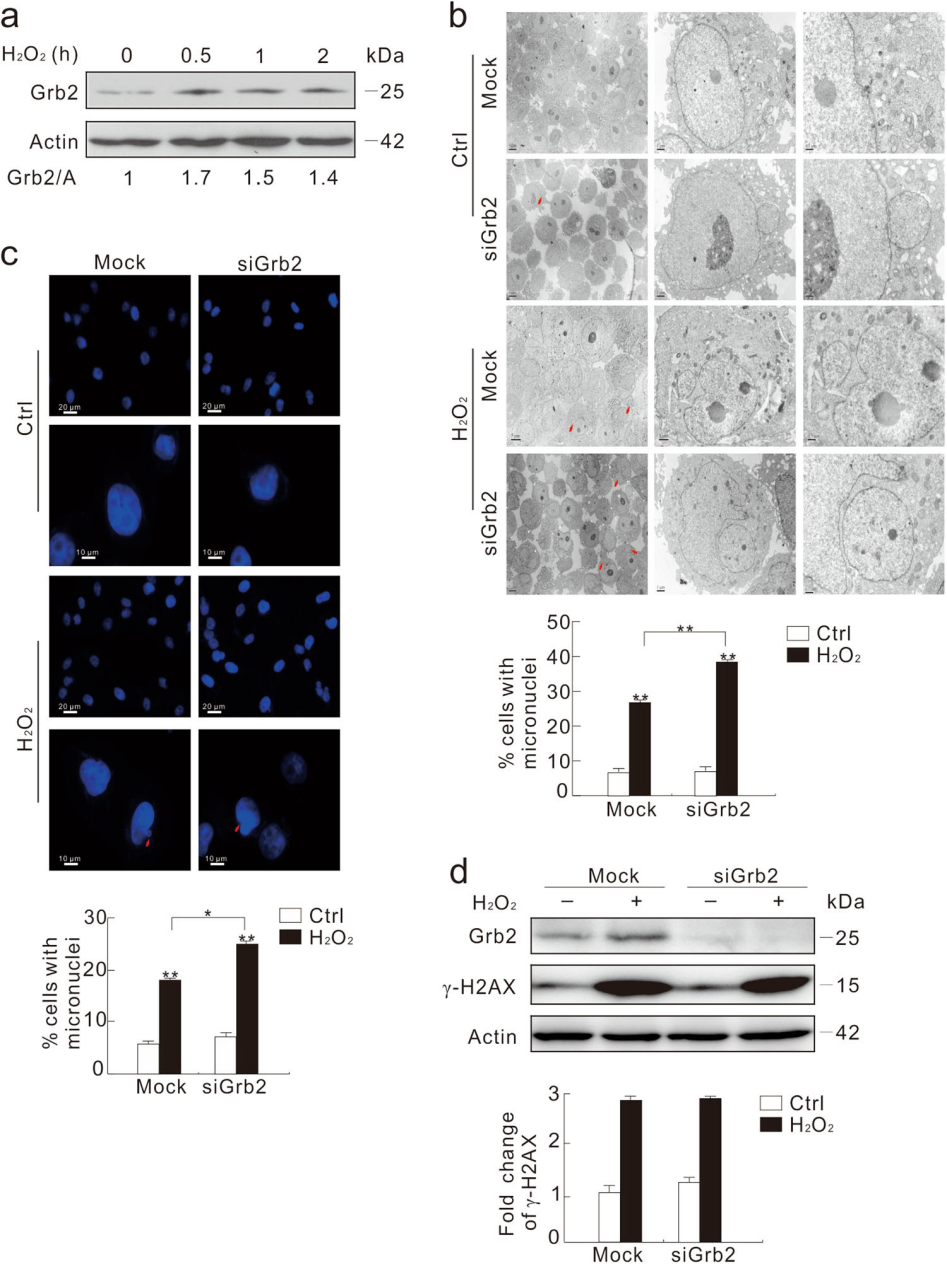
Depletion of Grb2 increases H_2_O_2_-induced formation of micronuclei. **a** HeLa cells were treated with 0.5 mM H_2_O_2_ for the indicated time, and cell lysates were subjected to immunoblotting with the antibodies indicated. **b**–**d** After transfection with the control (Mock) or Grb2 siRNA (siGrb2) for 48 h, HeLa cells were treated with 0.5 mM H_2_O_2_ for another 2 h. Transmission electron microscopy was performed and the percentage of micronuclei were analyzed (**b**). Cells were stained with DAPI, and observed with fluorescent microscope. The number of cells containing micronuclei was counted and at least 60 cells were included in each group (**c**). Cell lysates were subjected to immunoblotting with the indicated antibodies. The ratios of γ-H2AX to actin (A) were shown in graph (**d**). For histogram results, the data were presented as mean ± S.D., and analyzed by *T*-test. *P < 0.05 vs. control; **P < 0.01 vs. control. Similar experiments were repeated at least three times

Unexpectedly, Grb2 was increased in time- and dose-dependent manners under H_2_O_2_ treatment (Fig. 2a; Supplementary Fig. 2a). Since Grb2 silencing impaired DNA synthesis^2^, we speculated that Grb2 might play a role in maintaining nuclear stability. To prove the hypothesis, Grb2 in HeLa cells was knocked down and cells were observed with TEM. Although micronuclei were not significantly affected by the Grb2 deprivation alone, H_2_O_2_ markedly increased the frequency of micronuclei in the Grb2-depleted cells compared to the Mock-control (Fig. 2b, c). Notably, discontinuous envelope of micronuclei accompanying by small vacuoles was detected in the Mock-control group (Fig. 2b; arrow), but the nuclear envelope around micronuclei was intact, suggesting that degradation of micronuclei may be blunted in the Grb2-depleted cells. Since Grb2 loss did not augment the H_2_O_2_-induced level of γ-H2AX compared to the Mock-control (Fig. 2d), we assumed that Grb2 probably affected the repairing processes in response to DNA damage.

### Overexpression of dominantly negative (DN) Grb2 increased the H_2_O_2_-induced formation of micronuclei and inhibited the expression of Rad51

**Fig. 3:**
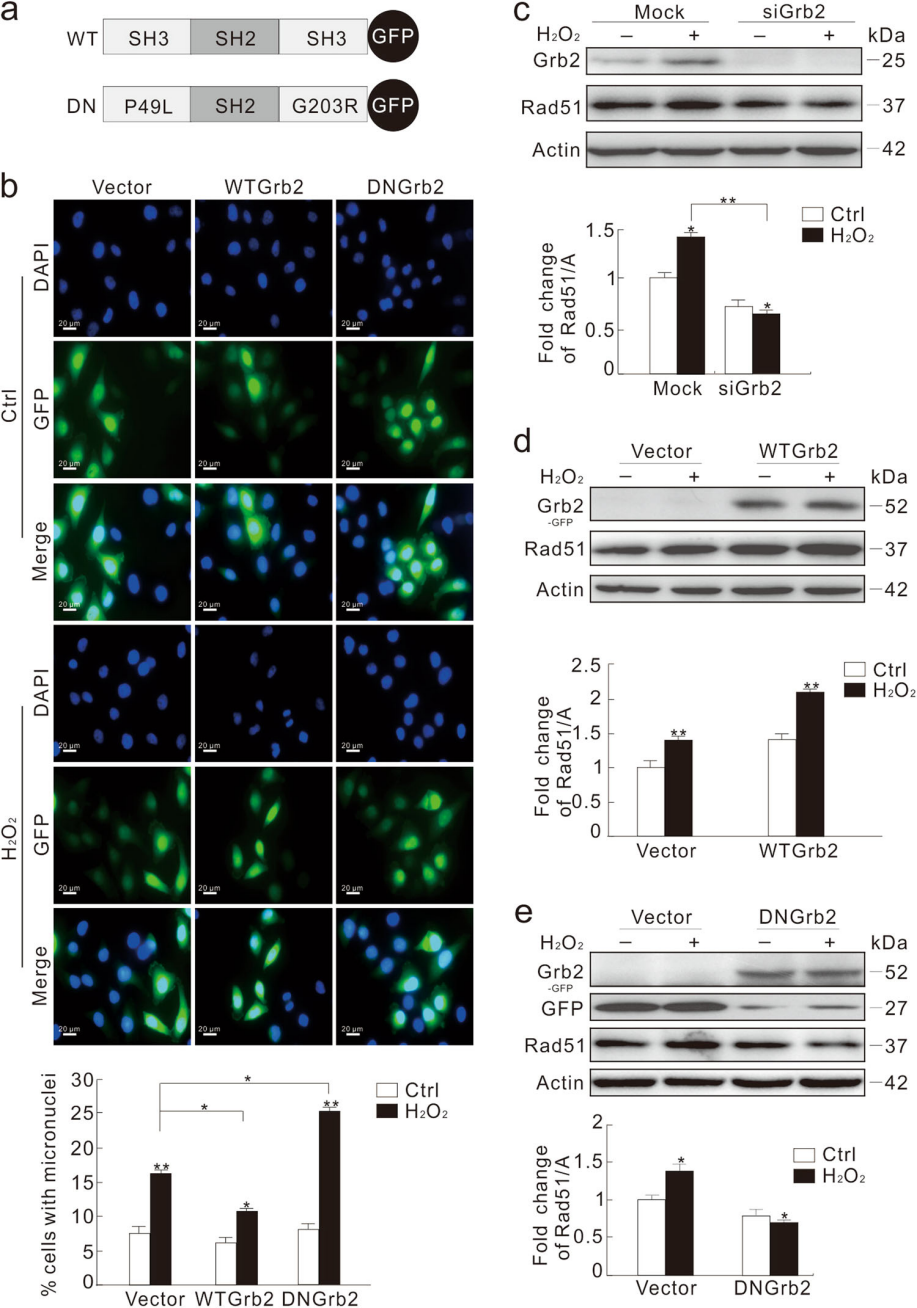
DN Grb2 attenuates the DNA homologous recombination repair in response to oxidative stress. **a** Schematic representation of the WT and mutant DN Grb2. **b** HeLa cells were transfected transiently with the GFP-vector, WT Grb2, or -DN Grb2 plasmids for 36 h, and then treated with 0.5 mM H_2_O_2_ for 2 h. The number of cells containing micronuclei was observed under microscope and shown in graph. **c** HeLa cells were transfected with the control (Mock) or Grb2 siRNA (siGrb2) for 48 h, and treated with 0.5 mM H_2_O_2_ for 2 h. Cell lysates were subjected to immunoblotting with the indicated antibodies. **d**, **e** HeLa cells were transfected transiently with the GFP-vector, WT Grb2 or DN Grb2 plasmids for 36 h, and treated with 0.5 mM H_2_O_2_ for 2 h. Cell lysates were analyzed by immunoblotting with the indicated antibody. For histogram results, the data were presented as mean ± S.D. and analyzed by *T*-test. *P < 0.05 vs. control; **P < 0.01 vs. control. Similar experiments were repeated at least three times

Previously generated constructs of Grb2, wild type Grb2 (WT Grb2), and P49L/G203R Grb2 (DN Grb2) were used to further examine the role of Grb2 in micronuclei formation (Fig. 3a). DN Grb2 serves as the loss-function-mutant to link its upstream and downstream molecules^31^. Overexpression of WT Grb2 decreased the H_2_O_2_-induced micronuclei frequency, whereas overexpression of DN Grb2 increased the formation of micronuclei compared to the vector-control with or without H_2_O_2_ treatment (Fig. 3b; Supplementary Fig. 2b, c).

Rad51 is critical for homology directed repair pathway^28–30^, and its loss impairs formation of DSB repair complex and chromosomal stability^30^. While H_2_O_2_ stimulated Rad51 expression in the Mock-control cells, Grb2 depletion reduced the level of Rad51 by 30%, which was decreased further upon H_2_O_2_ treatment (Fig. 3c). Overexpression of WT Grb2 increased the expression of Rad51 (Fig. 3d), whereas DN Grb2 with two mutated SH3 domains (Fig. 3a) decreased the level of Rad51 (Fig. 3e). In addition, the foci of Rad51 were increased in the WT Grb2-transfected cells, but decreased in the DN Grb2-overexpressed cells (Supplementary Fig. 2d). Since Grb2 silencing or DN Grb2 decreased Rad51 expression, micronuclei formation in the Rad51-depleted cells was examined. Different from Grb2 depletion, Rad51 loss alone obviously increased micronuclei frequency (Supplementary Fig. 3a, b), which was significantly increased in Grb2- and Rad51-deprived cells (Supplementary Fig. 3c). Given that Grb2 deprivation remarkably inhibited the H_2_O_2_-dependent Rad51 expression, we speculated that Grb2 loss mainly prevented cells from undergoing the HR repair process.

### Grb2 is involved in the interaction between PTEN and Rad51

**Fig. 4:**
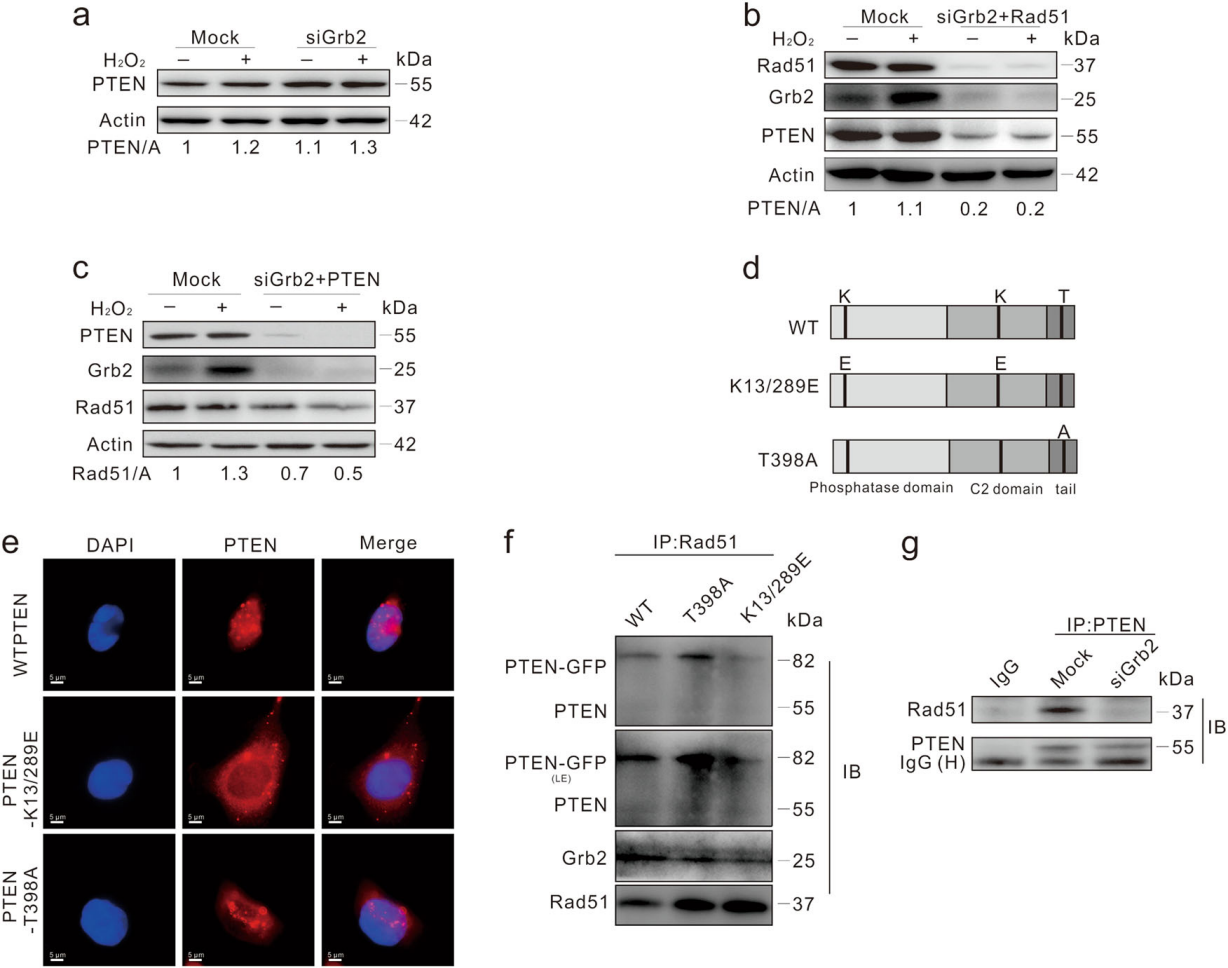
PTEN binds to Rad51. **a**–**c** HeLa cells were transfected with indicated siRNAs for 48 h, and treated with 0.5 mM H_2_O_2_ for 2 h. Cell lysates were subjected to immunoblotting with the indicated antibodies. **d** Schematic representation of the WT and mutant PTEN. **e** HeLa cells were transiently transfected with the WT and mutated PTEN for 36 h, stained with DAPI and PTEN antibodies, and observed with immunofluorescence microscope. **f** HeLa cells were transfected with the plasmids expressing PTEN for 36 h. Equal amounts of cell lysates prepared from transfection samples were immunoprecipitated with the antibody to Rad51. Immunoprecipitates and lysate inputs were then blotted with indicated antibodies. **g** HeLa cells were transfected with the control (Mock) or Grb2 siRNA (siGrb2) for 48 h, equal amounts of cell lysates were immunoprecipitated with the antibody of PTEN or IgG. Immunoprecipitates were then blotted for PTEN or Rad51. Similar experiments were repeated at least three times. L-Exp long time exposure

Recently, Grb2 loss was shown to inhibit the function of PTEN^3^, which mediated Rad51 expression by facilitating its transcription^4^. We found that PTEN remained undisturbed in Grb2-deprived cells (Fig. 4a), whereas Grb2/Rad51 depletion markedly reduced PTEN expression (Fig. 4b). Moreover, Rad51 silencing inhibited H_2_O_2_-induced upregulation of PTEN (Supplementary Fig. 3d). Considering this, we also explore whether Grb2 regulates Rad51 expression and maintains nuclear stability through PTEN^32^. Although Grb2/PTEN double deprivation failed to further decrease Rad51 expression compared to Grb2 silencing alone (Figs. 3c and 4c), micronuclei frequency was markedly increased in the Grb2/PTEN-depleted cells even in the absence of H_2_O_2_ (Supplementary Fig. 3e). Compared to the Mock-control, the foci of Rad51 were decreased in Grb2- or PTEN-deprived cells (Supplementary Fig. 3f). While the loss of Grb2 significantly reduced the expression of breast related cancer antigen 1 (BRCA1), a protein also involved in HR repair process (Supplementary Fig. 3g).

Since subcellular location of PTEN affects its function in DDR, different mutants including cytoplasm- (PTEN-K13/289E) and nuclear-localized (PTEN-T398A) PTENs^32,33^, were constructed, and their distribution pattern was examined using fluorescence microscope (Fig. 4d, e) and confocal microscopy (Supplementary Fig. 4a). Consistent with a previous finding, PTEN was observed in the immunoprecipitates of Rad51 (Fig. 4f)^10^. Although both PTEN-K13/289E and PTEN-T398A interacted with Rad51, much more PTEN-T398A was found in Rad51 immunoprecipitates, indicating that PTEN interacts with Rad51 in nucleus. We also found that Grb2 deprivation significantly decreased the interaction (Fig. 4g). Knockdown of Rad51 alone decreased the expression of both Grb2 and PTEN, whereas PTEN loss only reduced the level of Grb2 (Supplementary Fig. 4b).

### Grb2 interacts with both PTEN and Rad51

**Fig. 5:**
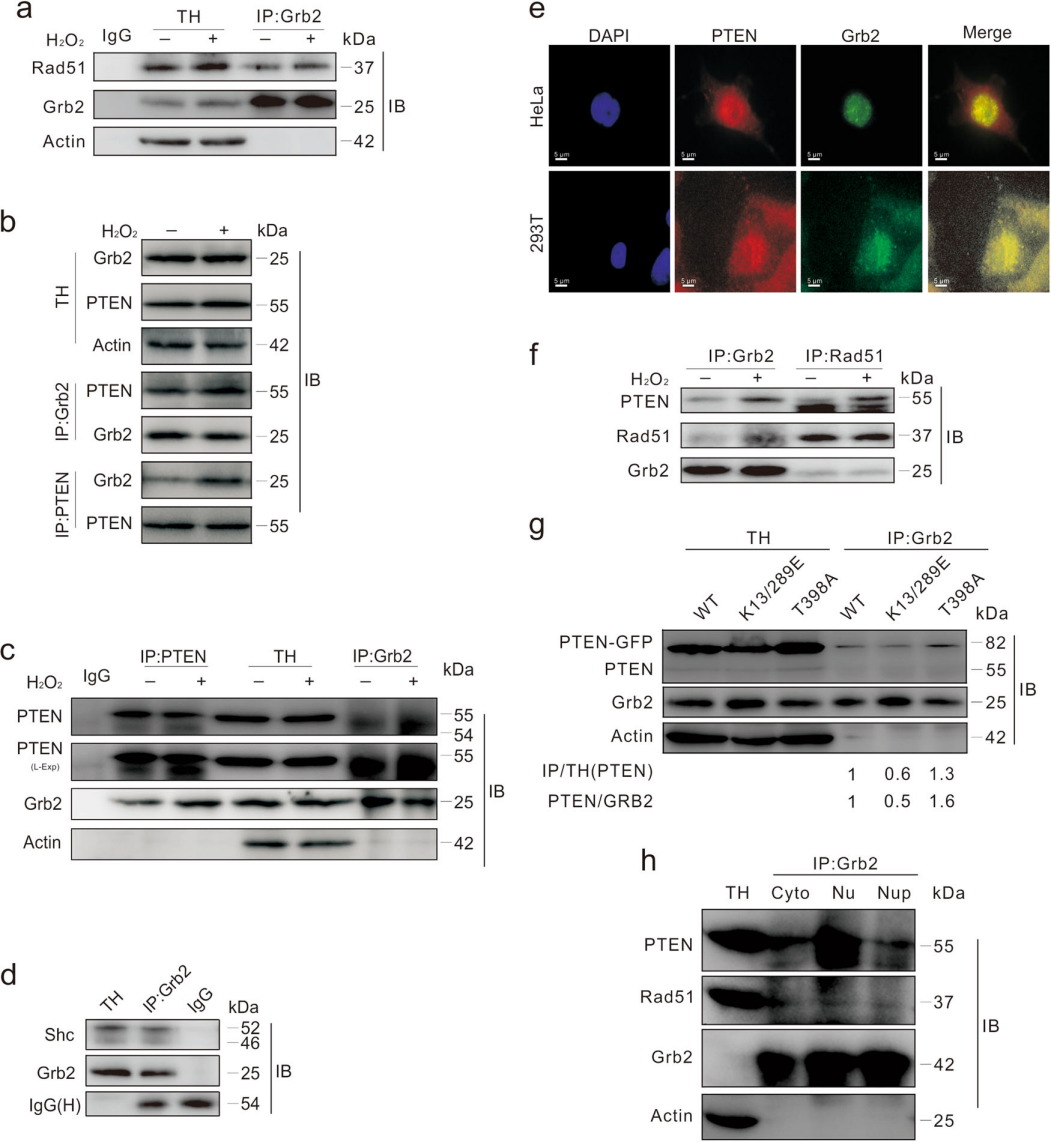
Grb2 binds to Rad51 and PTEN. **a** HeLa cells were exposed to 0.5 mM H_2_O_2_ for 2 h, and equal amounts of cell lysates were immunoprecipitated with the antibody to Grb2. **b**, **c** HEK293T or HeLa cells were exposed to 0.5 mM H_2_O_2_ for 2 h, and equal amounts of cell lysates were immunoprecipitated with the antibody of Grb2 or PTEN. **d** Equal amounts of HeLa cell lysates prepared were immunoprecipitated with the antibody of Grb2 or IgG. Samples were then blotted with indicated antibodies. **e** HEK293T cells and HeLa cells were stained with the Grb2 (green) and PTEN (red) antibodies following treatment with 0.5 mM H_2_O_2_ for 2 h. Similar experiments were repeated at least three times. **f** HeLa cells were exposed to 0.5 mM H_2_O_2_ for 2 h, and equal amounts of cell lysates were immunoprecipitated with the antibody of Grb2 or Rad51. **g** HeLa cells were transfected with the plasmids expressing PTEN mutants for 36 h. Equal amounts of cell lysates were prepared and immunoprecipitated with the antibody to Grb2. The ratios of PTEN in the immunoprecipitated group to GRB2 in the immunoprecipitated group were presented under the blots. **h** The total homogenate (TH), cytoplasm (Cyto), nuclear fraction (Nu) or nuclear precipitation fraction (Nup) of HeLa cells were extracted. Equal amounts of samples were immunoprecipitated with the antibody of Grb2. Similar experiments were repeated at least three times

The above results suggested that Grb2 and PTEN or Grb2 and Rad51 might have close relationships. To examine this, Grb2 was immunoprecipitated to explore its interaction with Rad51, and Rad51 was found to co-immunoprecipitate with Grb2 (Fig. 5a). Results of reciprocal immunoprecipitation revealed that Grb2 interacted with PTEN in HEK293T and HeLa cells (Fig. 5b, c), and the association was enhanced in the presence of H_2_O_2_ (Fig. 5c). By using the Rad51 antibody after chemical cross-linking, a band of relatively high molecular weight (ca. 119 kDa; approximate to the sum of molecular weights for Rad51, PTEN, and Grb2) was observed in immunoblotting at the same location as the Rad51, Grb2, and PTEN antibodies (Supplementary Fig. 4c). Since Shc is a binding partner of Grb2^34^, the detected Shc in the immunoprecipitates of Grb2 was used as the positive control (Fig. 5d). The results of immunoprecipitations and the data of co-localization of PTEN and Grb2 indicated that a considerable portion of PTEN and Grb2 traffic into the same subcellular compartment (Fig. 5e).

### Nuclear-associated Grb2 interacts with PTEN and Rad51

Grb2 can bind to both PTEN and Rad51 (Fig. 5f), and preferentially to nuclear-localized PTEN (Fig. 5g), suggesting the existence of interaction between Grb2 and PTEN in nucleus. Subcellular fractionation and immunoprecipitation with nuclear lysates were performed to confirm the binding of Grb2 to PTEN. Both PTEN and Rad51 were found in Grb2 immunoprecipitates of cytoplasm (Cyto), nuclear soluble fraction (Nu), and of insoluble fraction (NuP; supposed to be mainly a chromatin component)^10^, and PTEN was detected in Nu (Fig. 5h). These results indicated that Grb2 binds to either or both of PTEN and Rad51 in nucleus, and their interactions may be essential for the HR repair pathway and maintaining nuclear stability.

### Grb2 regulates subcellular localization of PTEN

**Fig. 6:**
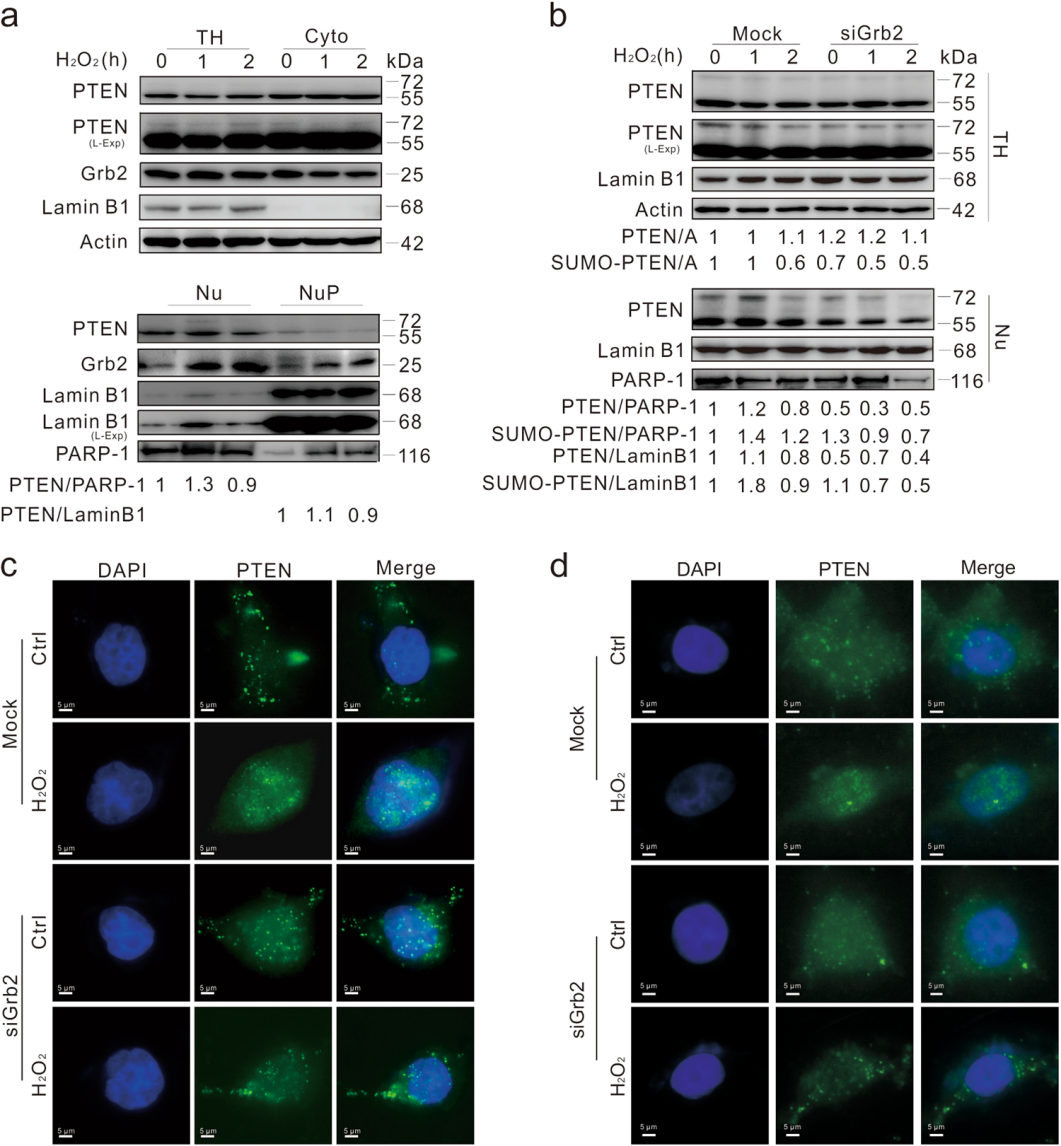
Depletion of Grb2 prevents PTEN from translocating into nucleus. **a** After treatment with 0.5 mM H_2_O_2_, TH, Cyto, Nu, and the Nup were extracted from HeLa cells and analyzed by immunoblotting with the antibodies indicated. **b** After 48 h transfection with the control (Mock) or Grb2 siRNA (siGrb2), HeLa cells were exposed to 0.5 mM H_2_O_2_ for up to 2 h, and then TH, and Nu were extracted and analyzed by immunoblotting with the antibodies indicated (Nu: soluble plus insoluble Nu). **c**, **d** HeLa cells were transfected with the control (Mock) or Grb2 siRNA (siGrb2) for 48 h, and treated with 0.5 mM H_2_O_2_ (C: 1 h; D: 2 h) before immunofluorescence analysis of PTEN localization. Similar experiments were repeated at least three times

Since PTEN accumulated in nucleus under genotoxic stress^32,35^, its subcellular distribution under H_2_O_2_ stimulation was examined. Consistently, we found that H_2_O_2_ induced nuclear accumulation of PTEN and stimulated its nuclear translocation in a time-dependent manner, with a visible amount of Grb2 appearing in Nu (Fig. 6a). Unexpectedly, a relatively larger amount of Grb2 was found in NuP, which appeared to be increased by H_2_O_2_ (Fig. 6a). Compared to the Mock-control, less PTEN was found in nuclei of Grb2-silenced cells at both the 1 and 2 h time points upon H_2_O_2_ challenge (Fig. 6b–d). Results from subcellular fractionation revealed that much less nuclear-localized PTEN was found in DN Grb2-transfected cells compared to the WT Grb2-overexpressed cells (Supplementary Fig. 5a, b). Consistent with that found previously^32^, PTEN was sumoylated and the SUMO-modified PTEN appeared in nucleus regardless of genotoxic stress (Fig. 6a, b; Supplementary Fig. 5c). Nevertheless, H_2_O_2_ induced less SUMOylated PTEN at the 2 h time point (Fig. 6a), while Grb2 loss reduced both the nuclear-localized and SUMOylated PTENs (Fig. 6b), which have MWs of 55 and 72 kDa, respectively. Although Grb2 ablation did not inhibit expression of the unmodified PTEN, it reduced the SUMOylated PTEN in total homogenate and nuclear lysates, whereas H_2_O_2_ reduced both PTENs in Grb2-depleted cells (Fig. 6b). Notably, H_2_O_2_ increased the nuclear-localized PTENs in the Mock-control at the 1 h time point, but Grb2 loss decreased the PTEN level in nuclear lysates (Fig. 6b), when both PARP and LaminB1 were used as the loading control and the nuclear marker. Here, PARP-1 and LaminB1 were used as the markers for Nu and NuP, respectively^36^. Since the nuclear-localized PTEN is critical to maintain genomic stability^32^, Grb2 loss likely impaired nuclear stability at least partially through reducing the nuclear-PTEN and Rad51.

### Nuclear-localized PTEN reduces micronuclei induced in DN Grb2-transfected cells

**Fig. 7:**
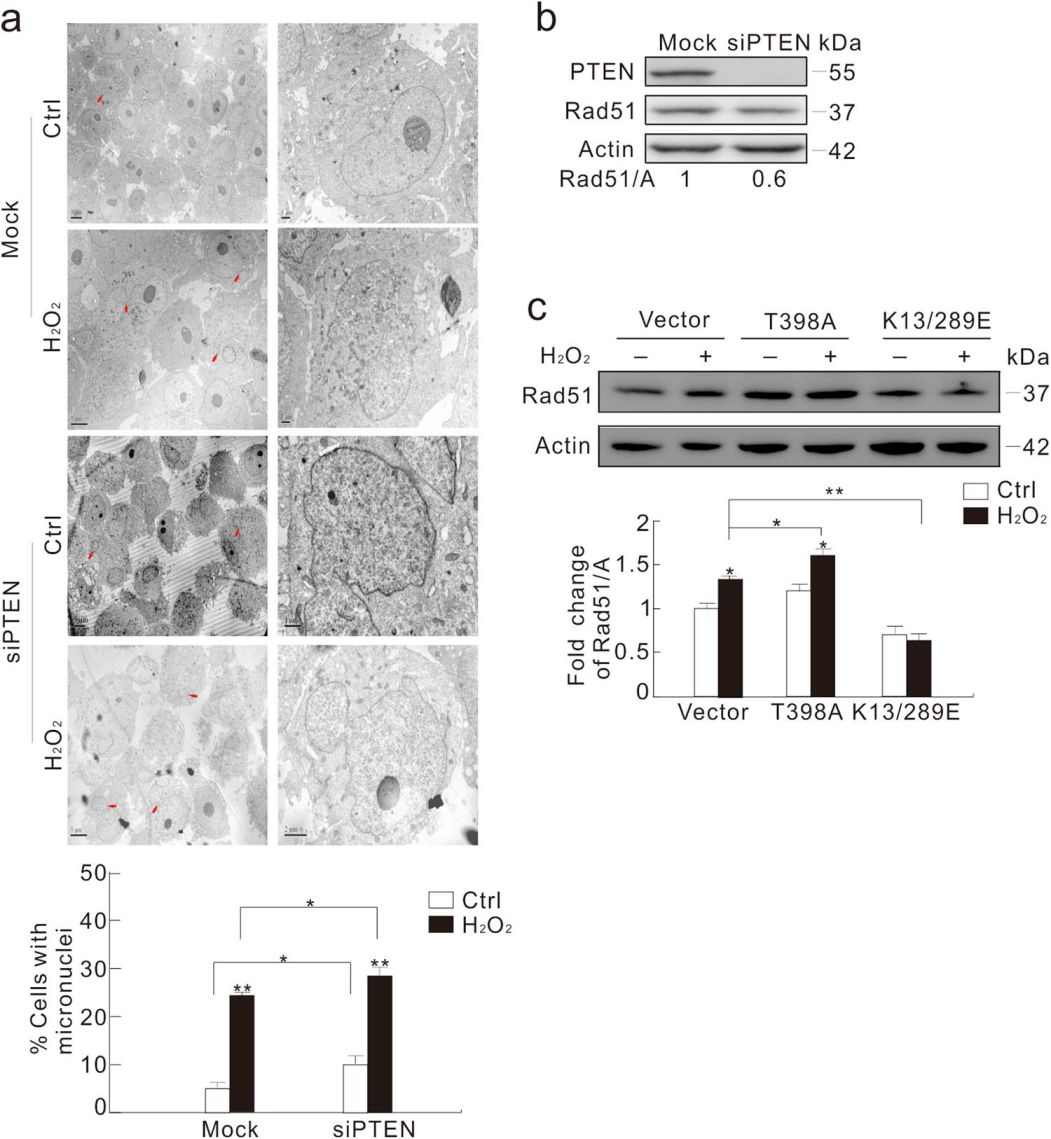
PTEN affects micronuclei frequency and Rad51 expression. **a**, **b** HeLa cells were transfected with the control (Mock) or the PTEN siRNAs for 48 h. Transmission electron microscopy was performed and the frequency of micronuclei were analyzed, and at least 60 cells were included in each group (**a**), cell lysates were subjected to immunoblotting with the indicated antibodies (**b**). **c** HeLa cells were transfected with the plasmids carrying WT, T398A, or K13/289E PTEN. After 36 h, cells were treated with 0.5 mM H_2_O_2_ for 2 h, and cell lysates were detected by immunoblotting with the indicated antibodies. The ratio of Rad51 to actin was shown in the graph. For histogram results, the data were presented as mean ± S.D. and analyzed by T-test. *P < 0.05 vs. control; **P < 0.01 vs. control. Similar experiments were repeated at least three times

Nuclear-localized PTEN played an essential role in maintaining genomic stability^4,9,32^, and we also found that PTEN loss accrued micronuclei upon H_2_O_2_ stimulation (Fig. 7a). Unlike Grb2 silencing, knockdown of PTEN alone increased the frequency of both micronuclei and abnormal nuclei concurring with a 40% decrease of Rad51 (Fig. 7a, b). However, PTEN loss failed to increase the γ-H2AX level upon H_2_O_2_ treatment, similarly to that found under Grb2 silencing (Supplementary Fig. 5d). Blurred micronuclei membrane in PTEN-depleted cells revealed increase in nuclear damage and failure in eliminating lesion (Fig. 7a; arrow).

**Fig. 8:**
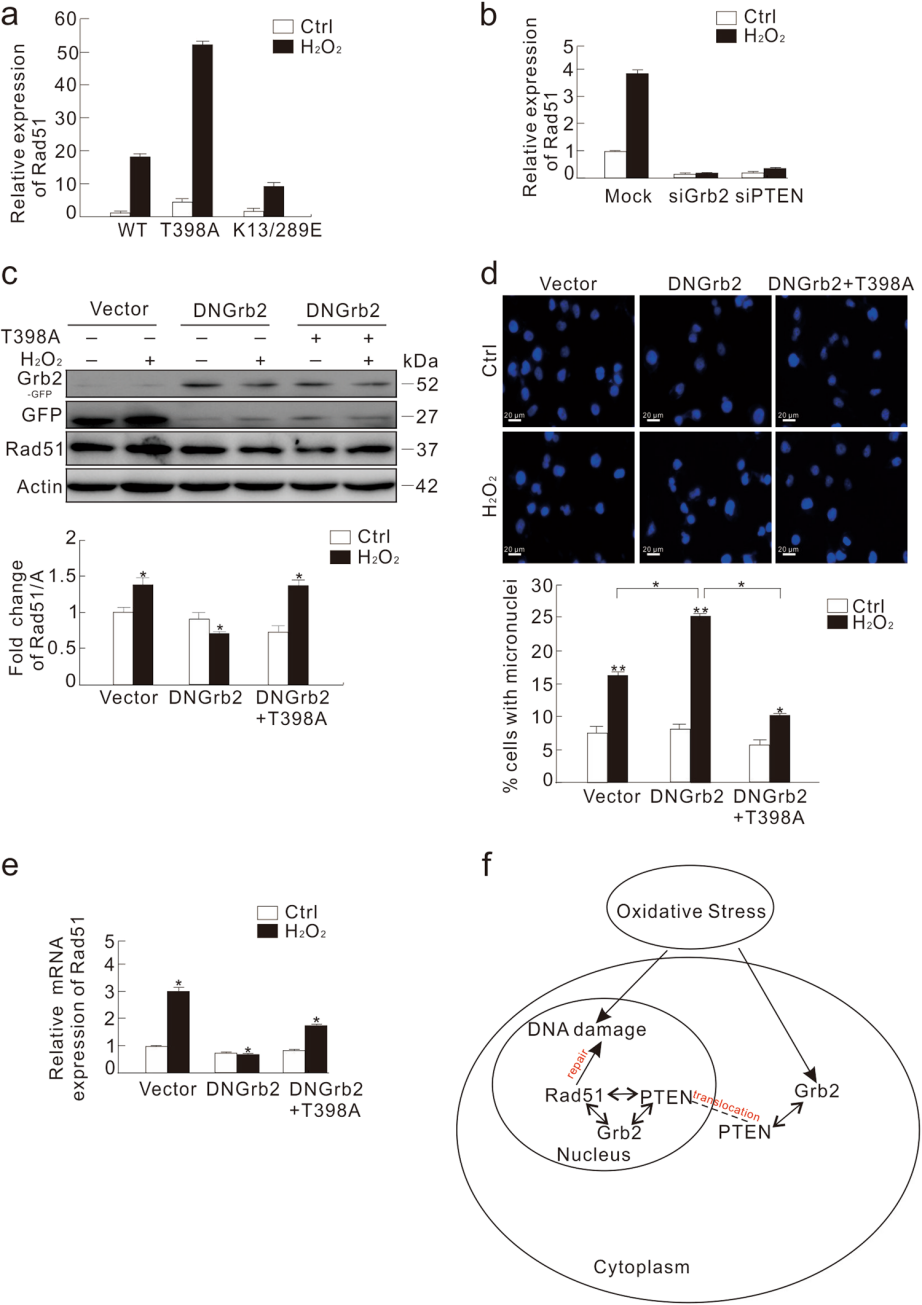
Overexpression of nuclear-located PTEN reduces H_2_O_2_-induced micronuclei formation in DN Grb2-transfected cells. **a** HeLa cells were transfected with the plasmids carrying WT, T398A, or K13/289E PTEN. After 36 h, cells were treated with 0.5 mM H_2_O_2_ for 2 h, and the total RNA was extracted, reversed, and detected by real-time PCR. **b** HeLa cells were transfected with siRNA of the control (Mock), Grb2 or PTEN. After 48 h, the cells were either left untreated or stimulated with 0.5 mM H_2_O_2_ for 2 h. Relative mRNA expression of Rad51 was presented in graph. **c**–**e** HeLa cells were transiently transfected with DN Grb2 alone or plus the T398A PTEN for 36 h, split, and treated with 0.5 mM H_2_O_2_ for 2 h. Cell lysates were blotted with the indicated antibody. The ratio of Rad51 to actin (A) was shown in the graph (**c**). Cells were fixed, stained with DAPI and observed with fluorescent microscope. The number of cells containing micronuclei was counted and at least 60 cells were included for each group (**d**). Relative mRNA expression of Rad51 was presented in graph (**e**). For histogram results, the data were presented as mean ± S.D. and analyzed by *T*-test. *P < 0.05 vs. control; **P < 0.01 vs. control. **f** Schematic mechanism of Grb2 and PTEN in the oxidative stress-induced DDR. Similar experiments were repeated at least three times

To further investigate the role of nuclear-localized PTEN in DNA damage^37^, HeLa cells were transfected with either PTEN-T398A or PTEN-K13/289E. While the T398A mutant increased Rad51 expression, the K13/289E mutant decreased the level of Rad51 (Fig. 7c), indicating that nuclear-localized PTEN is critical for Rad51 expression. Since PTEN was reported to facilitate Rad51 transcription^4^, and its nuclear translocation was affected by Grb2, we investigated the cause for decreased Rad51 expression in Grb2-depleted cells. Using quantitative real time PCR (qPCR), we observed that K13/289E-transfected cells contained much less Rad51 mRNA compared to the WT or T398A mutant (Fig. 8a), indicating that nuclear-PTEN contributes to Rad51 transcription. Knockdown of either Grb2 or PTEN significantly inhibited the H_2_O_2_-dependent Rad51 transcription (Fig. 8b), whereas Grb2 loss showed no obvious impact on PTEN mRNA (Supplementary Fig. 6a). Contrary to WT Grb2, overexpression of DN Grb2 inhibited the H_2_O_2_-induced Rad51 and BRCA1 transcription (Supplementary Fig. 6b, c). Moreover, T398A mutant rescued the H_2_O_2_-induced (not the basal) Rad51 expression and the inhibited H_2_O_2_-dependent micronuclei formation in the DN Grb2-transfected cells (Fig. 8c–e). The transfecting efficiency was shown in Supplementary Fig. 6d. These observations suggested that nuclear-PTEN might function downstream of Grb2 to regulate the H_2_O_2_-induced Rad51 expression. Thus, Grb2 maintained the nuclear stability through interacting with PTEN and mediating its nuclear translocation, and consequently affected Ras51 expression to regulate the DNA repairing process.

## Discussion

Although Grb2 is generally regarded as a cytoplasmic protein and plays a passive role in signal transduction, it was recently found to control the activity of EGF receptor kinase prior to growth factor binding^38^, and the nuclear-localized Grb2 has been found in breast tumor tissues^39^. In this study, nuclear staining of Grb2 was observed in either HeLa or HEK293T cells, and the nuclear localization of Grb2 was confirmed by subcellular fractionation. Moreover, Grb2 was found to interact with either PTEN or Rad51, both playing important roles in maintaining nuclear stability^28,32^.

Notably, either Grb2 or Rad51 can interact with nuclear-localized PTEN, and their bindings in nucleus were confirmed by immunoprecipitation using nuclear lysates. Therefore, Grb2 likely exerts its direct function on DNA damage and repairing, in addition to bridging the RTKs and cytoplasmic signaling pathways. Although Grb2 is well known as a tumor-promoting protein^40^, it was also reported to facilitate the function of PTEN, which played a role in maintaining genomic stability^3^. In addition, Grb2 appeared to affect the nuclear translocation of PTEN since Grb2 depletion markedly reduced the nuclear content of PTEN. Unlike deprivation of either PTEN or Rad51, ablation of Grb2 alone failed to significantly increase micronuclei frequency in HeLa cells (Mock: 7.4%; siGrb2: 8.7%). In this case, although Grb2 loss decreased Rad51 expression, the total unmodified PTEN maintained relatively normal in Grb2-depleted cells, whereas the level of SUMOylated PTEN in total homogenate and nuclear lysates was somewhat high. Since combined depletion of Grb2 and PTEN increased micronuclei formation and failed to further reduce suppression of Rad51 expression, PTEN was likely required by Grb2 in maintaining nuclear stability upon H_2_O_2_ stimulation. Although H_2_O_2_ did not inhibit the expression of unmodified PTEN in Grb2-depleted cells, it caused more severe loss of both Rad51 and SUMOylated PTEN in nuclear lysates. Therefore, adequate amount of nuclear-localized Rad51 appeared to be critical for genomic stability, and Grb2 might play a more important role in maintaining nuclear stability in response to oxidative stress. However, we observed that depletion of Grb2 alone reduced Rad51 expression and significantly increased micronuclei formation in mouse fibroblast B82 cells (data not shown). Thus, Grb2 likely mediates nuclear stability in a cell type-dependent manner.

Micronuclei are small extra-nuclear bodies originated from damaged chromosome fragments or whole chromosomes during nuclear division^41^. We found that the H_2_O_2_-induced genotoxic stress resulted in accumulation of micronuclei, which is in agreement with a previous report^25^. Despite PTEN was known to maintain chromosomal integrity^32^, upstream mediators for nuclear localization of PTENs remained largely unknown. In agreement with a previous finding^4^, we also found that nuclear localization is essential for PTEN to exert its nuclear stabilizing function. More important, Grb2 was found to interact with PTEN, and the interaction was enhanced upon treatment with H_2_O_2_. Although the loss of Grb2 did not affect the protein level of PTEN, Grb2 appeared to regulate the expression of SUMOylated PTEN and nuclear translocation. Since SUMOylated PTEN was reported to be more important for genomic stability^32^, Grb2 may function upstream of PTEN in regulating nuclear stability under certain scenery. Based on the results from this study, we believe that Grb2 plays a regulatory role in nuclear translocation of PTEN, which is important for Rad51 expression. Although post-transcriptional modifications, such as ubiquitination and phosphorylation, have been reported to regulate subcellular localization of PTEN^33,42^, our findings revealed a previously unnoticed pathway to regulate subcellular location of PTEN.

Two principal pathways exist for DSB repair^14,15^. Rad51 is a key player in HR by forming complexes with other proteins^28–30^. The protein level of Rad51 is associated with HR activity, and the loss of which leads to inhibition of DSB repair^30^. Consistent with these reports, we found that deprivation of Rad51 alone caused nuclear instability. Since Grb2 loss markedly inhibited the level of H_2_O_2_-induced Rad51, and combined depletion of Grb2 and Rad51 increased micronuclei frequency, Grb2 may regulate nuclear stability through mediating the expression of Rad51. Surprisingly, we found that Rad51 could interact with either Grb2 or PTEN, while its loss reduced their protein levels. Therefore, coordination among the three proteins may exist to maintain nuclear stability. Notably, it appeared that all the proteins should be concomitantly downregulated to cause defective HR, at least in un-stimulated HeLa cells. Since the loss of Grb2 failed to decrease the expression of PTEN and to accumulate micronuclei, depletion of either PTEN or Rad51 led to increase in micronuclei frequency concurring with downregulation of Rad51/Grb2 or PTEN/Grb2. Given that Rad51 loss resulted in downregulation of both PTEN and Grb2, we speculated that interactions among the three proteins could be important for their own stability and nuclear integrity. Since silencing of either Grb2 or PTEN was found to regulate Rad51 expression at the transcriptional level in response to DDR, we assumed that a feed-forward or feed-back mechanism might exist to regulate the expression of these proteins depending on cell type and condition. Since Rad51 can bind to other proteins involved in maintaining genomic stability^28,29^, and both Grb2 and Rad51 can interact with PTEN in nucleus, we believe that Grb2 and PTEN may be components of the Rad51-formed complex for nuclear stability.

In summary, the findings presented here offer mechanistic insights into the connection between nuclear stability and Grb2 (Fig. 8f), an essential molecule for signaling transduction and endocytosis. Our data indicated that Grb2 can bind to both PTEN and Rad51, and functions upstream of PTEN to regulate the latter’s nuclear translocation and consequently affect the expression of Rad51 in controlling the DDR process.

## Materials and methods

### Cell culture and Western blot analysis

HeLa and HEK293T cells were grown in DMEM medium (HyClone; SH20022.01B) containing 10% fetal bovine serum (GIBCO; 16000) and antibiotics. Cells grown to 70–80% confluency prior to addition of H_2_O_2_ were treated in completed medium containing 10% serum. For transfection, cells grown to 80% confluency were transfected using Lipofectamine 2000 (Invitrogen) or Attractene (QIAGEN) according to the manufacturer’s protocol. After 36 h transfection, cells were split and cultured overnight prior to different treatments, immunoblotting, or fluorescence microscopy. For siRNA, cells were grown to 30–40% confluency in their respective media without antibiotics and transfected using DharmaFECT (Dharmacon; T2001 or T2002) according to the manufacturer’s instructions. After 48 h transfection, cells were split and cultured overnight prior to stimulations. Whole cell lysates were prepared with lysis using Triton X-100/glycerol buffer^43^, containing 50 mM Tris-HCl, 4 mM EDTA, 2 mM EGTA, and 1 mM dithiothreitol (pH 7.4), supplemented with 1% Triton X-100 and protease inhibitors, and separated on a SDS-PAGE gel (13 or 8%, according to the MWs of the proteins of interest), and transferred to PVDF membrane. Western blot assays were performed using appropriate primary antibodies and horseradish peroxidase-conjugated suitable secondary antibodies, followed by detection with enhanced chemiluminescence (Pierce Chemical). X-ray films and Tanon-5200 were employed to collect the chemiluminescence signals and the quantifications were carried out using densitometry.

### Subcellular fractionation

Chromatin and cytosolic/soluble extracts were obtained as previously described^44^ or using Nuclear and Cytoplasmic Extraction Reagents according to manufacturer’s instruction. Briefly, cell extracts were prepared in the harvest buffer (10 mM HEPES, 50 mM NaCl, 0.5 M sucrose, 0.1 M EDTA, 0.5% Triton X-100; pH 8.0) containing both protease inhibitors (1 mM dithiothreitol, 2 mg/ml pepstatin, 4 mg/ml aprotinin, and 100 mM PMSF) and phosphatase inhibitors (10 mM tetrasodium pyrophosphate, 100 mM NaF, and 17.5 mM *β*-glycerophosphate). The low-speed supernatant (500 g) containing the cytoplasmic proteins was collected and the nuclear extracts were prepared by vortexing the nuclei at 4 °C for 15 min in a buffer containing 20 mM HEPES (pH 7.9), 400 mM NaCl, 1 mM EDTA, 1 mM EGTA, 0.1% IGEPAL CA-630, and protease inhibitors. The extracts were mixed with the half volume of 3 × loading buffer and boiled for 10 min. The unsolvable precipitations were suspended by loading buffer and boiled for 30 min or lysed again with Triton X-100/glycerol buffer.

### Immunoprecipitation

Immunoprecipitation of PTEN, Rad51, or Grb2 was performed in HeLa and HEK293T cells. Equal amounts of the total homogenate, nuclear fraction extracted with Nuclear and Cytoplasmic Extraction Reagents or nuclear precipitation fraction were incubated with PTEN, Grb2, or Flag monoclonal antibody. The PTEN or Grb2 immunocomplexes were precipitated by protein A/G-Sepharose. For Western blotting, immunoprecipitates or cell lysates were resolved in SDS-polyacrylamide gels and then transferred onto a nitrocellulose filter. The blots were incubated with PTEN, Rad51 or Grb2 antibodies, and then with peroxidase-conjugated species-matched secondary antibodies.

### Chemical cross-linking

Cells were seeded into 100-mm dishes at 70–80% confluency and cultured overnight. Then cells were gathered and washed twice with cold PBS after the indicated treatment. After resuspending the pellet with PBS to 5 × 10^6^ cells/ml, 4% paraformaldehyde was added to the final concentration of 0.5%, and then cells were incubated for 30 min at 37 °C. Following addition of 2.5 M glycine (pH 3.0) to the final concentration of 125 mM and incubation at room temperature (RT) for 5 min, cells were centrifuged at 2000 rpm for 5 min. After washing twice with PBS, Triton X-100/glycerol buffer was added to acquire the whole cell lysates, which were then used in the following immunoprecipitation experiments. For the samples acquired after chemical cross-linking, the denaturation temperature was set at 70 °C instead of 96 °C, to avoid depolymerization of the complexes.

### Immunofluorescence microscopy

HeLa or HEK293T cells were split and grown on coverslips overnight before addition of H_2_O_2_ for the time indicated. Cells were fixed with freshly prepared 4% paraformaldehyde at RT for 12 min, incubated with the indicated antibodies, and stained with Alex Fluor 488 or 594 secondary antibodies. Images were captured using fluorescence microscopy.

### Confocal microscopy

Cells were split and grown on coverslips overnight before addition of WT PTEN, PTEN-K13/289E, or PTEN-T398A for the time indicated. Cells were fixed with freshly prepared 4% paraformaldehyde at RT for 12 min, incubated with the indicated antibodies, and stained with Alex Fluor 594 secondary antibodies. Images were captured using con-focal microscopy.

### Electron microscopy

Electron microscopy was performed as described^45^. Briefly, samples were washed three times with PBS, trypsinized, and collected by centrifuging. The cell pellets were fixed with 4% paraformaldehyde at 4 °C overnight, post-fixed with 1% OsO_4_ in cacodylate buffer at RT for 1 h and dehydrated stepwise with ethanol. The dehydrated pellets were rinsed with propylene oxide at RT for 30 min and embedded in Spurr resin for sectioning. Images of thin sections were observed under a transmission electron microscope (JEM1230, Japan).

### Reverse transcription and real-time PCR

The total cellular RNA was extracted using TRIzol reagent (Invitrogen; 15596–018) according to the manufacturer’s protocol, and the RNA integrity was confirmed by electrophoresis on ethidium bromide-stained 1% agarose gel. One microgram of the total RNA was reversely transcribed using PrimeScriptTM RT reagent Kit (TaKaRa; DRR037A). Real-time PCR was carried out using a SYBR real-time PCR kit (Sigma-Aldrich) in a Eppendorf Mastercycle EP (Eppendorf AG 22331; Hamburg). Primer sequences used for amplification were as follows: Rad51 upstream primer, 5′-ATG CCG TCG GAGAAG ACC-3′; downstream primer, 5′-TTA CAC TGA CAA TTT CAT CC-3′; PTEN upstream primer, 5′-TGG ATT CGA CTT AGA CTT GAC CT-3′; downstream primer, 5′-GGT GGG TTA TGG TCT TCA AAA GG-3′; BRCA1 upstream primer, 5′- GTC CAA AGC GAG CAA GAG-3′; downstream primer, 5′- CCT GTG CCA AGG GTG AAT-3′; *β*-actin upstream primer, 5′-GCC TGA CGG CCA GGT CAT CAC-3′; downstream primer, 5′-CGG ATG TCC ACG TCA CAC TTC-3′. Expression of *β*-actin was used as the internal control.

### Statistical analysis

The quantification analysis of immunoblots was carried out using densitometry. Statistical significance was analyzed using one-way ANOVA and the Student–Newman–Keuls post-hoc test. Results of multi-plates enzyme immunoassay and real-time PCR were shown as mean ± SD.

## Supplementary information

Supplementary Information.
