# Supplementary material for: Grb2 binds to PTEN and regulates its nuclear translocation to maintain the genomic stability in DNA damage response
Source: Cell Death Dis. 2019 Jul 18;10(8):546. doi: 10.1038/s41419-019-1762-3 (PMC6639399; doi:10.1038/s41419-019-1762-3)
Supplement: Supplementary file 1 — Supplementary Information. [file 41419_2019_1762_MOESM1_ESM.doc]

**Grb2 binds to PTEN and regulates its nuclear translocation to maintain the genomic stability in DNA damage response**

Running Title: Grb2 binds to PTEN to maintain genomic stability

Bolin Hou,^1,2,†^ Shanshan Xu,^1,2,†^ Yang Xu^3^, Quan Gao,^1,2^ Caining Zhang,^1,2^ Ling Liu^1^, Huanyi Yang,^4^ Xuejun Jiang^1^,* and Yongsheng Che^3^,*

**This Supplementary information includes:**

**Supplementary Figure: Figure 1-6**

**Supplementary Figure 1**


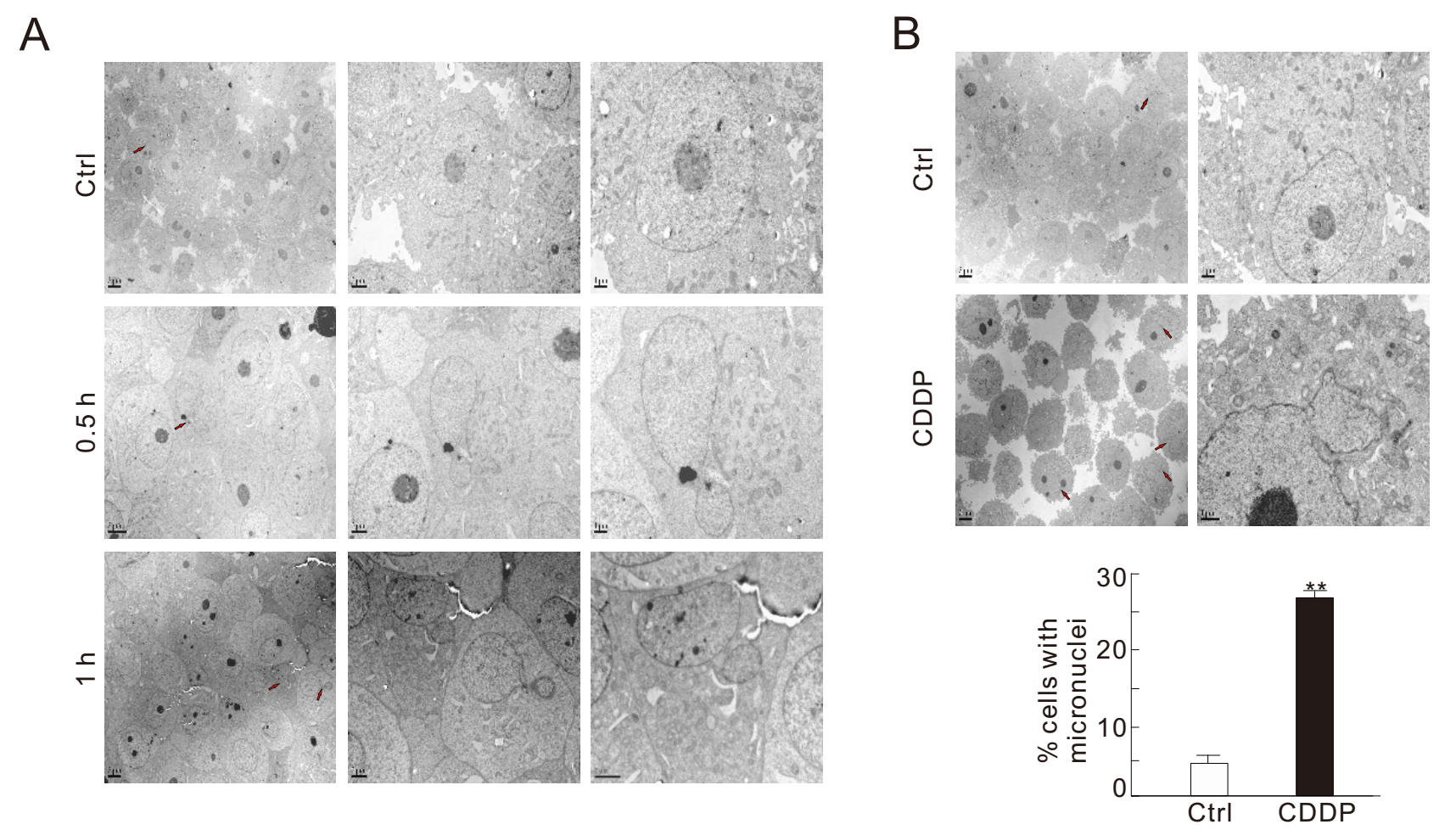


**Supplementary Figure 1: H_2_O_2_ induces micronuclei formation.** (**A** and **B**) Electron microscopy was performed with the vehicle (Ctrl) or H_2_O_2_ (0.5 mM; 0.5 and 1 h), or CDDP (50 μM) for 2 h, and at least 60 cells were included in each group. Similar experiments were repeated at least three times.

**Supplementary Figure 2**


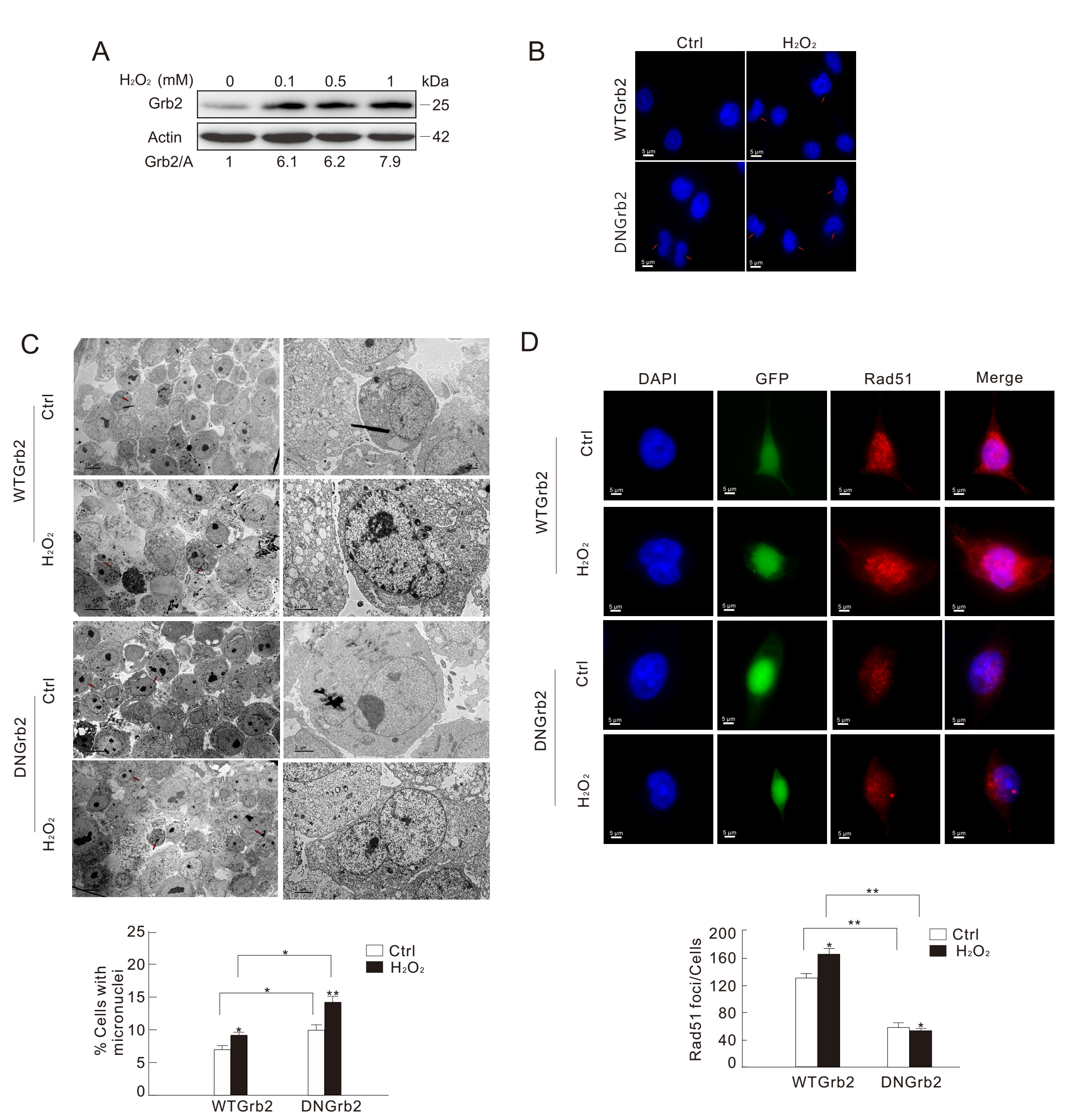


**Supplementary Figure 2: DN Grb2 increased the H_2_O_2_-induced micronuclei formation and inhibited the foci of Rad51.** (**A**) HeLa cells were treated with H_2_O_2_ (0-1 mM) for 2 h, and immunoblotting analysis was performed with the indicated antibodies. (**B**-**D**) HeLa cells were transiently transfected with the plasmids of WT or DN Grb2 for 36 h, cells were split onto coverslips (**B** and **D**) or 60 cm dishes (**C**) overnight, treated with 0.5 mM H_2_O_2_ for 2 h, stained with DAPI with or without the antibody of Rad51, and observed with fluorescent microscope (**B** and **D**), or acquired images via EM (**C**). The arrows indicate typical micronuclei. The number of cells containing micronuclei was counted and at least 30 cells were included in each group.

**Supplementary Figure 3**


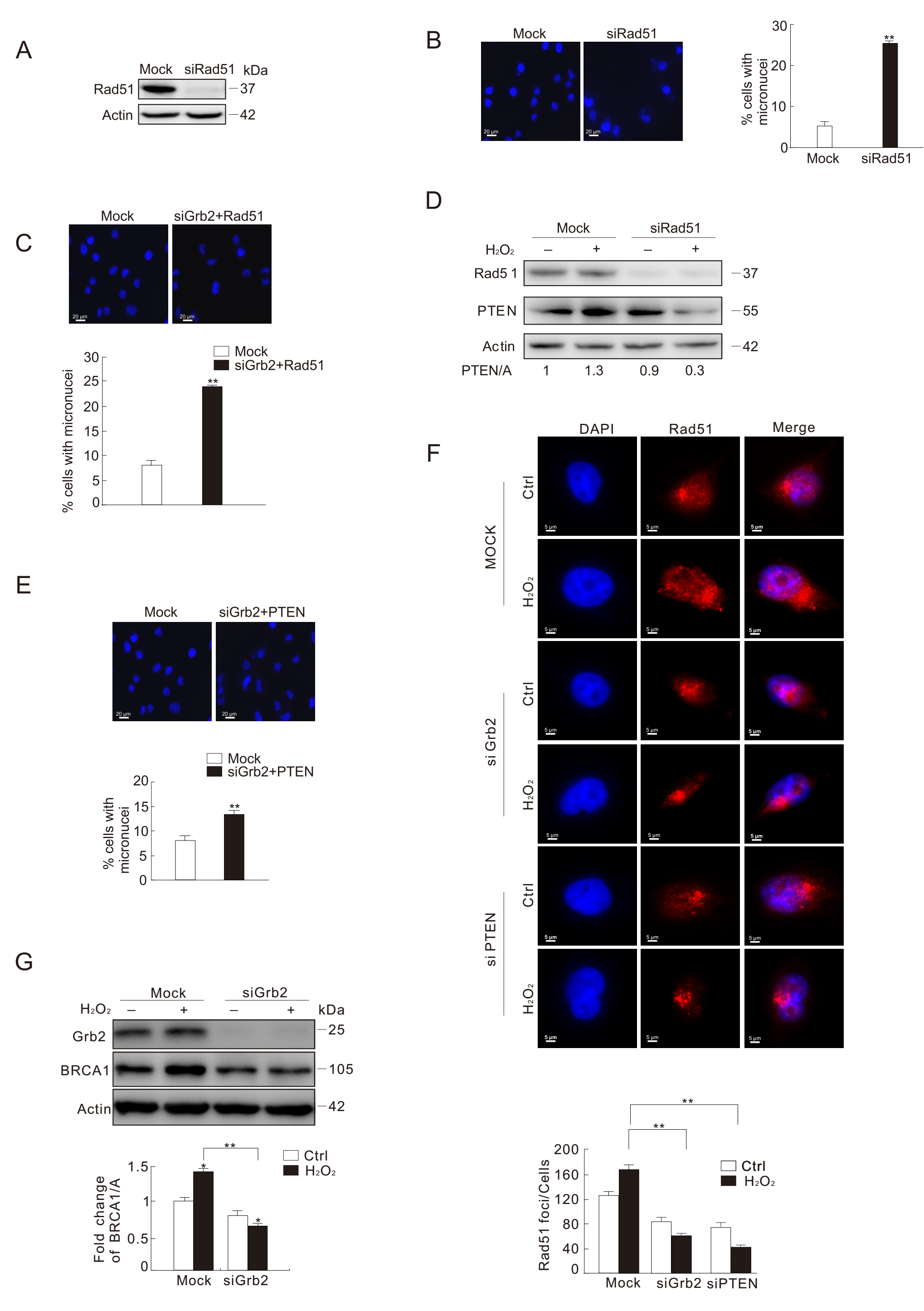


**Supplementary Figure 3: Knockdown of either Grb2 or PTEN reduced the foci of Rad51 and the expression of BRCA1.** (**A** and **B**) HeLa cells were transfected with the control (Mock) or Rad51 siRNA for 48 h. Cell lysates were analyzed by immunoblotting with the indicated antibodies (**A**). Transfected HeLa cells were split onto coverslips, stained with DAPI, and observed with fluorescent microscope (**B**). (**C** and **E**) HeLa cells were transfected with the indicated siRNAs for 48 h. The transfected HeLa cells were split onto coverslips, stained with DAPI, and observed with fluorescent microscope. The number of cells containing micronuclei was counted and at least 60 cells were included in each group. (**D**) HeLa cells were transfected with the indicated siRNAs for 48 h. After treatment with or without H_2_O_2_ (0.5 mM) for 2 h, cell lysates were detected by immunoblotting with the indicated antibodies. (**F**) HeLa cells were transfected with indicated siRNAs for 48 h, and treated with H_2_O_2_ (0.5 mM) for 2 h. Immunofluorescence was performed using the Rad51 antibody following treatment with H_2_O_2_ (0.5 mM) for 2 h. (**G**) HeLa cells were transfected with the control (Mock) or Grb2 siRNA (siGrb2) for 48 h, and treated with H_2_O_2_ (0.5 mM) for 2 h. Cell lysates were subjected to immunoblotting with the indicated antibodies. Similar experiments were repeated at least three times.

**Supplementary Figure 4**


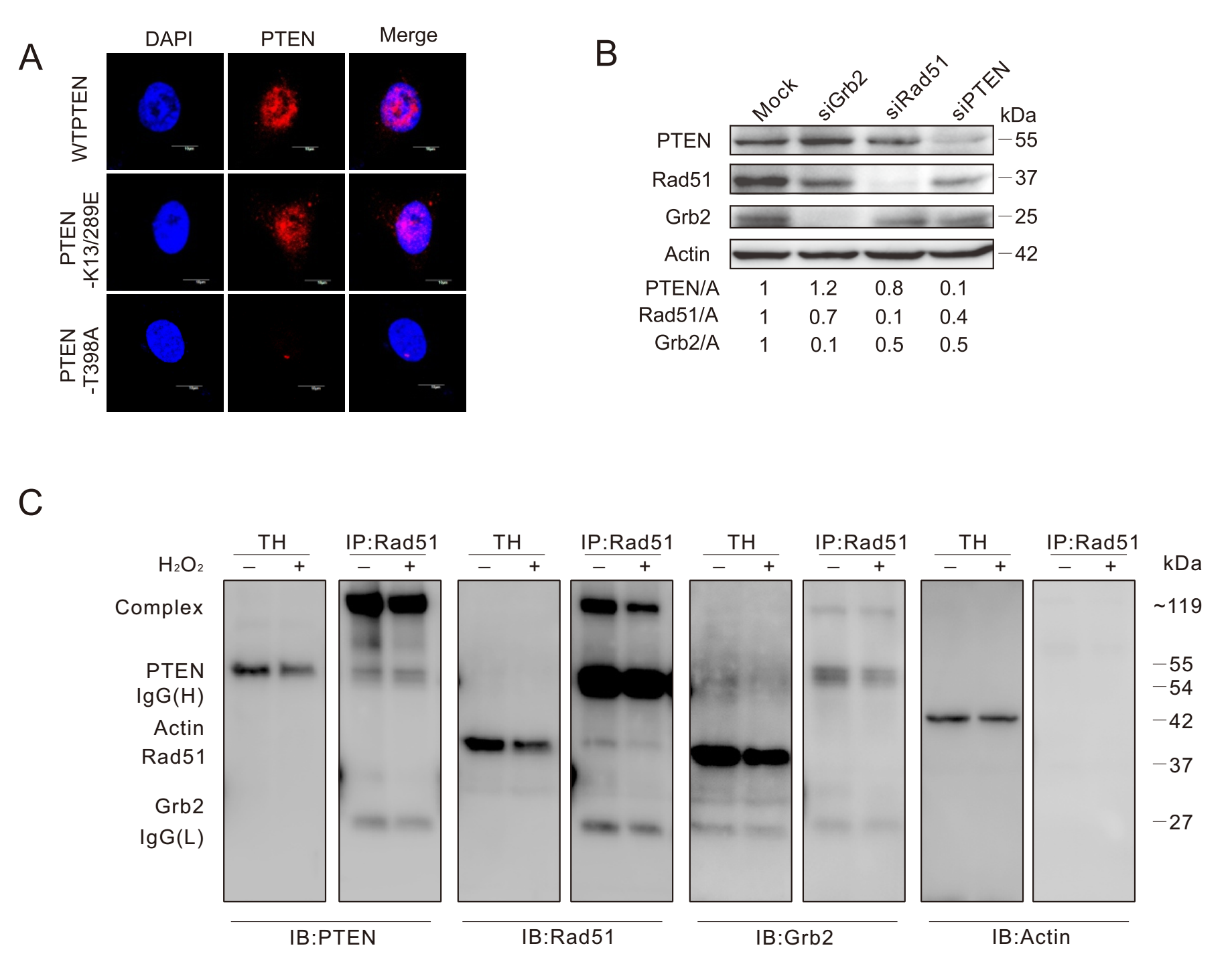


**Supplementary Figure 4: Grb2, PTEN, and Rad51 form a protein complex.** (**A**) HeLa cells were transiently transfected with the WT-Flag and mutated PTEN-Flag for 36 h, stained with DAPI and Flag tag antibody, and observed with confocal microscopy. Similar experiments were repeated at least three times. For histogram results, the data were presented as mean ± S.D. and analyzed by T-test. **P < 0.01 vs. control. (**B**) HeLa cells were transfected with the indicated siRNAs for 48 h. After treatment with or without H_2_O_2_ (0.5 mM) for 2 h, cell lysates were detected by immunoblotting with the indicated antibodies. (**C**) Following treatment with H_2_O_2_ (0.5 mM) for 2 h, HeLa cells were subjected to chemical cross-linking and immunoprecipitation with the Rad51 antibody. The inputs and immunoprecipitates were analyzed by immunoblotting with the antibodies indicated. Similar experiments were repeated at least three times.

**Supplementary Figure 5**


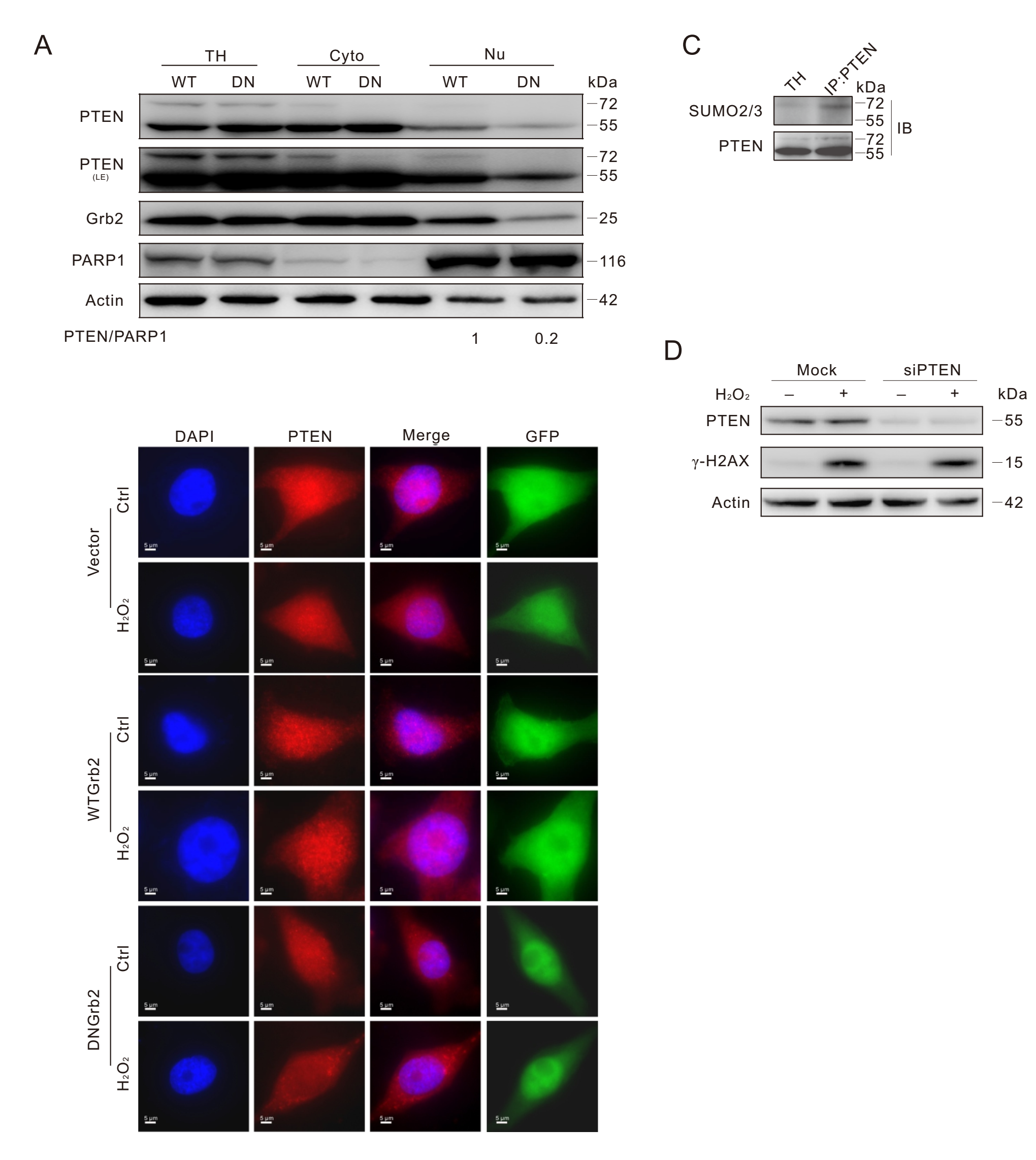


**Supplementary Figure 5: Overexpression of DN Grb2 reduces nuclear accumulation of PTEN.** (**A** and **B**) HeLa cells were transfected with WT Grb2 or DN Grb2 for 48 h, and TH, Cyto, and Nu were extracted and analyzed by immunoblotting with the antibodies indicated (**A**). Cells were left either untreated, or stimulated with H_2_O_2_ (0.5 mM) for 2 h, stained with PTEN antibody, and observed with fluorescent microscope (**B**). (**C**) PTEN was immunoprecipitated from HeLa cell lysates and immunoblotted with the PTEN antibody, and stripped and reprobed with the antibodies to Sumo2/3. (**D**) HeLa cells were transfected with the indicated siRNAs for 48 h. After treatment with or without H_2_O_2_ (0.5 mM) for 2 h, cell lysates were detected by immunoblotting with the indicated antibodies. Similar experiments were repeated at least three times.

**Supplementary Figure 6**


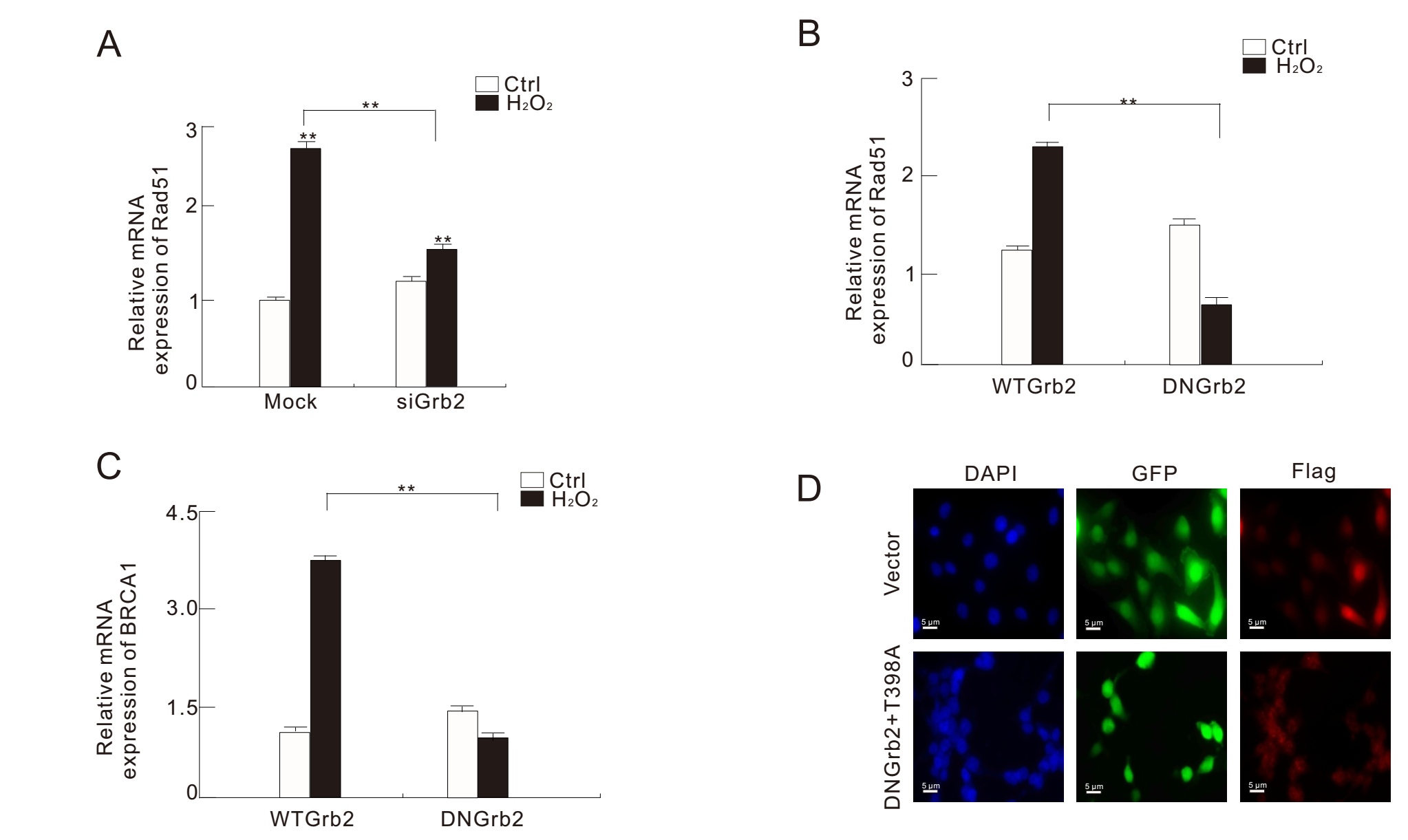


**Supplementary Figure 6: DN Grb2 blocks the transcription of Rad51 and BRCA1.** (**A**) HeLa cells were transfected with siRNA of Grb2. After 48 h, cells were either left untreated or stimulated with H_2_O_2_ (0.5 mM) for 2 h. Relative mRNA expression of PTEN was presented in graph. (**B**) HeLa cells were transfected with the WT Grb2 and DN Grb2. After 36 h, cells were treated with H_2_O_2_ (0.5 mM) for 2 h. Relative mRNA expression of Rad51 was presented in graph. (**C**) HeLa cells were transiently transfected with the indicated plasmids. Transfection efficiency was observed with fluorescent microscope. (**D**) HeLa cells were transfected with the WT Grb2 and DN Grb2. After 36 h, cells were treated with H_2_O_2_ (0.5 mM) for 2 h. Relative mRNA expression of BRCA1 was presented in graph. Similar experiments were repeated at least three times.
